# Extracting samples of high diversity from thematic collections of large gene banks using a genetic-distance based approach

**DOI:** 10.1186/1471-2229-10-127

**Published:** 2010-06-24

**Authors:** Marco Pessoa-Filho, Paulo HN Rangel, Marcio E Ferreira

**Affiliations:** 1 Departamento de Biologia Celular, IB - Universidade de Brasília (UnB) Campus Universitário Darcy Ribeiro, Asa Norte, CEP 70910-900, Brasília DF, Brazil; 2 Embrapa Cerrados, PO Box 08223, CEP 73 310-970 Planaltina, DF, Brazil; 3 Embrapa Arroz e Feijão, PO Box 179, CEP 75 375-000, Santo Antonio de Goiás, GO, Brazil; 4 Embrapa Recursos Genéticos e Biotecnologia, Genetics Lab, PO Box 02372, CEP 70 770-917 Brasília, DF, Brazil

## Abstract

**Background:**

Breeding programs are usually reluctant to evaluate and use germplasm accessions other than the elite materials belonging to their advanced populations. The concept of core collections has been proposed to facilitate the access of potential users to samples of small sizes, representative of the genetic variability contained within the gene pool of a specific crop. The eventual large size of a core collection perpetuates the problem it was originally proposed to solve. The present study suggests that, in addition to the classic core collection concept, thematic core collections should be also developed for a specific crop, composed of a limited number of accessions, with a manageable size.

**Results:**

The thematic core collection obtained meets the minimum requirements for a core sample - maintenance of at least 80% of the allelic richness of the thematic collection, with, approximately, 15% of its size. The method was compared with other methodologies based on the M strategy, and also with a core collection generated by random sampling. Higher proportions of retained alleles (in a core collection of equal size) or similar proportions of retained alleles (in a core collection of smaller size) were detected in the two methods based on the M strategy compared to the proposed methodology. Core sub-collections constructed by different methods were compared regarding the increase or maintenance of phenotypic diversity. No change on phenotypic diversity was detected by measuring the trait "Weight of 100 Seeds", for the tested sampling methods. Effects on linkage disequilibrium between unlinked microsatellite loci, due to sampling, are discussed.

**Conclusions:**

Building of a thematic core collection was here defined by prior selection of accessions which are diverse for the trait of interest, and then by pairwise genetic distances, estimated by DNA polymorphism analysis at molecular marker loci. The resulting thematic core collection potentially reflects the maximum allele richness with the smallest sample size from a larger thematic collection. As an example, we used the development of a thematic core collection for drought tolerance in rice. It is expected that such thematic collections increase the use of germplasm by breeding programs and facilitate the study of the traits under consideration. The definition of a core collection to study drought resistance is a valuable contribution towards the understanding of the genetic control and the physiological mechanisms involved in water use efficiency in plants.

## Background

A core collection is defined as a sub-sample of accessions that represent, with the lowest possible level of redundancy, the genetic diversity of a cultivated species [1]. Core collections are used to facilitate the access of potential users to samples of small sizes, representative of the genetic variability contained within the gene pool of a specific crop [2]. The ever increasing number and size of germplasm collections in gene banks around the world makes it necessary to establish procedures to limit the size of core collections. The reason for this is that, quite often, some of them are greater than expected by potential users, such as breeding programs. The eventual large size of a core collection perpetuates the problem it was originally proposed to solve, i.e., the definition of a group of accessions with enough genetic variability and with a sample size amenable to be used by the client.

Usually, core collections can be seen from two perspectives: from a taxonomist point of view, in which case rare alleles should be represented in any core collection; or from the point of view of a plant breeder, in which case the main requirement for conservation would be to maximize the representation of the genetic diversity of the species for practical purposes [1]. Here, we would argue that a core collection, from a breeder's perspective, should also be thematic, i.e., it should be composed of a sample of unique accessions which represent the genetic diversity of a cultivated species for a specific trait. As a result, in addition to the classic core collection concept, which has its justified attributes [2], several core collections should be also developed for a specific crop, each focused on a specific theme (i.e., for rice, a core collection for drought tolerance, cold tolerance, blast resistance, etc.) and composed of a limited number of accessions, with a size manageable by breeding programs.

Different criteria have been defined so far for the analysis of genetic diversity in order to compose a core collection. The majority of the proposed strategies vary in their methods by either the stratification of the reference collection in groups that are genetically closer when examined according to some criteria, or by taking a straight sample of the accessions that will make up the core collection according to a specific methodology. Stratification can be based on criteria which include morpho-physiological and agronomical traits [3], geographical parameters [4], biochemical traits [5], or molecular data [6]. Stratified random sampling methodologies include random sampling with no regard to group origin, sampling proportionate to the size of the groups, or proportionate to the natural logarithms of the size of the groups that are composed after the first stage of stratification [7], or may even be based on more concrete data on allelic composition of the reference population [8] or based on genetic distance estimated by biochemical or molecular markers [9-13].

As molecular marker information becomes more available, their usage as a criterion for establishing core collections has become increasingly appealing. Molecular marker information reflects changes that occur directly in the DNA, while morphological evaluations reflect changes in the phenotype, which are largely defined by more than one genetic locus, and which may have a strong environmental influence on the expression of the trait being analyzed. Thus, accessions that present similar phenotypes may not necessarily possess a close genetic relationship [11]. Until recently, molecular marker genotyping techniques which could attend the high demands of sample characterization in germplasm banks were time and resource consuming [14]. With the development of new medium or high scale genotyping techniques, molecular characterization became more accessible. [15] and [11] made use of data generated by isoenzymes and RAPD techniques, respectively, as tools to generate the information necessary for the delimitation of core collections of potato, cocoa and pepper plants. In addition, ideal sampling techniques to establish the core collections have also been examined. Over the past few years, proposals of methodologies for establishing core collections that use data generated by the application of molecular markers in conjunction with the morpho-agronomical characterization of the accessions have been given more attention [10,12,16,17].

The present study presents and evaluates a genetic-distance based sampling methodology to develop collections using rice as a model, and a thematic collection as a source of accessions to compose the core. The gene banks of rice in several countries are relatively extensive and usually harbor a large number of accessions. As a consequence, the core collections developed are usually also sizeable, not particularly attractive to be explored by breeding programs. The breeder is not always willing to evaluate and explore the diverse accessions that compose a core collection, unless there is known variation for specific traits directly related to the needs of his elite populations. The sampling methodology for core collections discussed here could be of use from this perspective.

The methodology is based on pairwise genetic distances between accessions that compose a thematic collection for a trait of interest, in order to select a core collection which will meet the following standard requirements: the maintenance of a pre-defined minimal proportion of alleles of the total collection, and a sample size that facilitates its practical usage by breeding programs. At first, we suggest the selection of a group of accessions with genetic variability for a specific breeding target. Then, accessions that are genetically more distant between themselves in this group are selected. Measurements of allele richness retained on samples of different sizes are calculated and plotted to indicate the most adequate size of the core collection. DNA polymorphism data which was generated by genotyping upland rice accessions from EMBRAPA's germplasm bank using microsatellite markers was used for testing this strategy. The results were compared with other sampling strategies recently proposed for core collection design using the same molecular data generated for the thematic reference collection.

## Methods

### Plant material and microsatellite genotyping using multiplex panels

Detailed information regarding plant material, protocols for the extraction of DNA, PCR conditions for multiplex panels, in addition to other genetic analysis related to the accessions which were used as the starting point for this study can be found in [18].

The trait selected for the development of a thematic core collection was drought tolerance in rice. Initially, some criteria had to be established to select a thematic collection of accessions with genetic variability for the trait to serve as the starting point to establish the core collection for drought tolerance. Drought tolerance is a complex, quantitative trait, particularly important for rice cropped under aerobic or rainfed upland growth conditions, where water is provided to the plant by natural precipitation. Rice germplasm adapted to these growth conditions are particularly found in the *japonica *group [19]. Most of the mapping populations designed to map drought tolerance QTL, derived from *indica *x *japonica *crosses, usually detect favorable alleles for drought-resistance traits contributed by *japonica *lines [20]. Therefore, a thematic collection of rice accessions was developed which was composed of *japonica *varieties, including: (a) Brazilian rice landraces adapted to drought prone environments, (b) accessions cultivated under upland or aerobic conditions in the tropics, (c) modern cultivars bred for drought tolerance, (d) accessions of *japonica *rice which presented some information of cultivation in non-irrigated conditions.

A collection of 699 accessions was selected for the analysis. Genotype multilocus profiles of each accession based on 16 microsatellite markers were used to estimate pairwise genetic distances between the accessions [18]. One hundred and fifty-one accessions with *indica *or mixed genetic background were initially excluded from the initial collection. Out of the remaining 548 accessions, a total of 485 tropical *japonica *accessions genotyped were treated as the thematic collection for this study.

### Statistical analysis

Genetic distance values were based on the coefficient of shared allele distance, estimated by the ratio between the sum of the proportions of common alleles between two accessions (Ps) for all loci and twice the number of tested loci [21,22]. Genetic distances were finally obtained following the parameter [(-ln (Ps)] on the web-based Genetic Distance Calculator [23].

Estimates of the total number of alleles, observed heterozygosity (Ho), gene diversity (GD) and polymorphism information content (PIC) were calculated using the program Power Marker v. 3.25 [24]. The expected gene diversity was calculated based on the unbiased estimator formed by the ratio between the expected heterozygosity (1 - Σ*i *p*i *^2^) and the factor (2n)/(2n - 1); being p*i *the frequency of the *i*th allele for each locus and n the number of analyzed samples [25]; the coefficient of endogamy f was estimated according to the method of moments [26]. The program Powermarker was also used to estimate linkage disequilibrium between the microsatellite loci, through the coefficients *D*' [27] and *r *^2 ^[28] and also to estimate the significance of the values of linkage disequilibrium between pairs of loci.

### Selection of a thematic core collection for drought tolerance based on genetic distance data

A diagonal matrix of pairwise genetic distances between the 485 accessions of *japonica *rice was used as the initial file for the calculations of the program Corex - Core Extractor (Ferreira et al., unpublished). Initially, the pairwise comparisons with maximum genetic distances observed among the 117.370 possible combinations were ranked. With the objective of defining the best size of a core collection, sub-samples of different sizes, varying from 25 to 300 accessions, were extracted from the thematic collection of 485 accessions. For each sample size, the extraction of the accessions showing the maximum genetic distance was carried out with a minimum of 100 repetitions. Allele richness ([Number of alleles/Total number of alleles] × 100) was estimated for each sample size using the program. A graph plotting the sample size of the core collection versus the allele richness of each sample (i.e., the percentage of alleles from the thematic collection retained on each core collection) was used to determine the sample size that would bring retention of at least 80% of the allelic richness of the thematic collection, following the original principles defined by [29]. The resulting core collection (CC _corex_) was compared to a collection of the same size obtained by random sampling of accessions (CC _random sampling_), also with 100 repetitions. The sampling was carried out without replacing the chosen accessions. This strategy was defined as the Corex method (Ferreira et al., unpublished). The results were compared to MStrat [30] and Powercore [17] procedures, which are based on the M Strategy (M for Maximization), proposed by [8], aiming to maximize the allelic representation in the core collection. The same data that was used with the Corex method were utilized for the M Strategy. The resulting core collections (CC _corex_, CC _random sampling_, CC _Mstrat _and CC _Powercore_) based on the different sampling strategies were then compared.

The genetic structure of the core collection obtained by the Corex method (CC _corex_) was analyzed using the software Structure version 2.1 [31]. Analysis of genetic distances and grouping were initially used as a reference to detect possible signs of structuring, which would suggest a potential presence of subpopulations in the sample. A burn-in period of 100,000 iterations followed by a running time of 1,000,000 iterations was used. Five independent analyses for each *K *were carried out, with the values for *K *varying from 1 to 15. The criteria for detecting the most likely *K *was the Δ*K*, an *ad hoc *quantification related to the change of a second order in the logarithmic probability of the data in relation to the number of groups inferred by the Structure algorithm [32]. An accession was included in a specific group inferred by the program if at least 70% of its genomic value, measured according to its inclusion coefficient (which varies from 0 to 1), was estimated as pertaining to that group. Average values of *F*_ST _for the inferred groups were calculated through the program Powermarker. The correlation between the groups defined by Structure and groups defined by analysis of genetic distance and grouping by Neighbor-Joining was estimated using the coefficient of Pearson. The comparison between average values of gene diversity for each loci between different collections was carried out using the Student's t test, Wilcoxon's signed-rank test, and a Bayesian approach implemented to the R platform (algorithm TEST_h_DIFF, available at http://www.ucl.ac.uk/tcga/software/index.html) [33].

### Comparison of different methodologies for building a core collection using quantitative data as a measure of diversity

The core collection obtained using the Corex algorithm (CC _corex_) was evaluated in the field for drought tolerance in Gurupi, State of Tocantins, Brazil, in upland growing conditions with irrigation control. A triple Latin Square 10 × 10 experimental design, with seed sowing density of 70 - 80 seeds/m, 35 cm of spacing between furrows and plots composed of four furrows of 3 meters each (sampled area of 1,4 m ^2^) was used in the field. The experiment was submitted to adequate soil humidity (minimum of -0.025 MPa at 15 cm deep) until the culture was established. A system of controlled irrigation based on daily tensiometer readings was implanted 30 days after seedling emergence, with a cycle of irrigation of 38 mm being applied when the water potential of the soil, at 15 cm of depth, reached -0.025 MPa. The treated plots were submitted to drought stress receiving only 50% of the water supplied to the control plots until the end of the plant cycle. The irrigation was manipulated by using a self-propelled system of horizontal bars that are 40 meters wide with a mechanical elevator to regulate the distance between the spray nozzle and the top of the plant (IrrigaBrasil, system 75/GB). The horizontal bars housed 26 units of 16mm spray nozzles, model Senninger, with an outflow pressure of 41.2 m ^3^/hectare. Data was collected for the following traits: Leaf Surface Temperature, Number of Days to Flowering, Plant Height (in cm), Grain Yield, Spikelet Sterility, the Weight of 100 Grains, the Number of Tillers and the Number of Panicles. ANOVA of each trait was carried out using the GENES program [34]. Although several different traits were evaluated, for the purposes of the present study only the data concerning the "Weight of 100 Grains" was considered when comparing different methodologies for establishing a core collection. An analysis of the remaining traits and agronomic evaluation of the core collection for drought tolerance is provided elsewhere [35]. Core sub-collections (CSC) of the CC _corex _using the four methodologies (Corex, MStrat, PowerCore, and random sampling) were constructed, also using their shared allele distance values as input data. The criteria for the construction were the same for the four methodologies, as previously described. Bartlett's test for homogeneity between variances for the trait "Weight of 100 Grains" was used to evaluate the differences between variances in the accessions of the core collection and the accessions in the core sub-collections, based on phenotypic field data. The comparison between average values of gene diversity for each loci between different core sub-collections was carried out using the Student's t test, Wilcoxon's signed-rank test, and a Bayesian approach implemented to the R platform (algorithm TEST_h_DIFF, available at http://www.ucl.ac.uk/tcga/software/index.html) [33].

## Results

### Definition of a core collection of landraces of rainfed upland rice using genetic distance data

**Figure 1:**
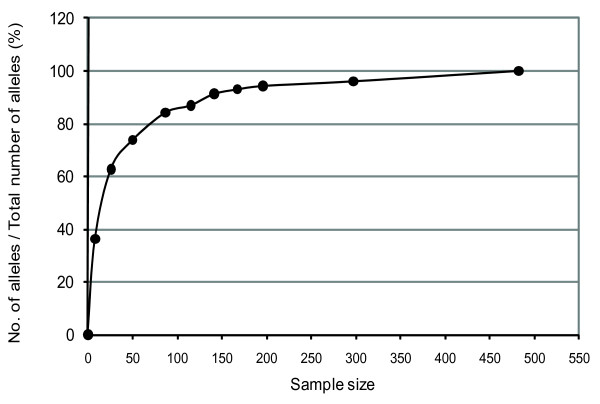
**Relationship between the percentage of alleles retained from the thematic collection and the sample size of thematic core collections**.

Core collections with sizes that varied from 25 to 300 accessions were constructed using the Corex method. For each analysis, with the objective of establishing a core collection of a specific size, only the samples which were present in all of the repetitions (i.e., with 100% inclusion when the 100 repetitions of each collection were accounted for) were considered to calculate the total number of alleles for each sample size and for the calculations of gene diversity (*GD*). A graph which plots the percentage of different alleles versus the number of samples included in each sample size was obtained (Figure 1).

As a result, a group of accessions composed of 87 rice varieties, retaining a minimum of 80% of the total number of alleles in the thematic collection, was defined (CC _corex_). These accessions presented a total of 193 alleles (out of 229 alleles for all the 485 accessions that were analyzed in the thematic collection), representing 84.3% of the total number, and an average of 12 different alleles for each locus (14 for all of the 485 accessions). Gene diversity estimates had an average value of 0.764 and were significantly different (*p *≤ 3.052 × 10 ^-5 ^in Wilcoxon'signed-rank test; *p *≤ 6.309 × 10 ^-5 ^in Student's t test; *p *= 0 in the Bayesian method) from the average of 0.667 obtained when the thematic collection of 485 accessions of upland rice was considered (Table 1).

**Table 1:** Comparison of core collections obtained from a thematic collection of 485 rainfed upland rice landraces by different methodologies (Corex, random sampling, MStrat and Powercore)

	Number of Alleles	Size %	n	*GD*
Thematic collection	229	10	485	0.667
				
CC_corex_	193 (84.3%)	17.94	87	0.764
CC_random sampling_	167 (72.9%)	17.94	87	0.647
CC_MStrat_	196 (85.6%)	10.51	51	0.739
CC_Powercore_	229 (100%)	16.90	82	0.745

The results demonstrated that the selection of the accessions which were more genetically distant, using the Corex methodology, extracted a core collection composed of 87 accessions with approximately 18% of the size of the thematic collection (485 accessions) (Table 1). This core collection still maintained approximately 84% of the alleles initially detected in the thematic collection.

### Genetic structure of the thematic core collection

The accessions of the core collection (CC _corex_) which were defined using the Corex methodology were submitted to an analysis based on the Structure program. It was observed that there was an increase in the estimated probability values as the potential *K *values also increased. For CC _corex_, the value of Δ*K *had its highest value at *K *= 3. Nevertheless, the number of subgroups found in the thematic collection without the accessions with *indica *background was indicated as *K *= 2, as described by [18]. The overall value of *F*_ST _was lower in comparison to the value of the thematic collection which was composed of 485 accessions and two inferred clusters (*F*_ST _= 0.128 for the core collection, *F*_ST _= 0.156 for the thematic collection). Effects of inbreeding were evident in CC _corex _as noticed by the high values of *F*_IS _and *F*_IT _(0.955 and 0.961, respectively). Values of *F*_ST _between clusters were also lower than those of the thematic collection, demonstrating that a smaller degree of differentiation existed between the inferred groups of CC _corex _(varying between 0.120 and 0.138), although it was still significant. AMOVA, using the results of the grouping analysis of genetic distances as a reference, indicated that 11.5% of the variation was caused by the differences between the two groups, with the rest of the variation being caused by differences within the groups. The AMOVA for the thematic collection showed that 8.7% of the variation was caused by differences between the two inferred groups. Seven accessions (8%) were identified as admixed in CC _corex _- that is to say they do not present coefficients of inclusion in a determined group above 70%.

### Genetic diversity and linkage disequilibrium

**Figure 2:**
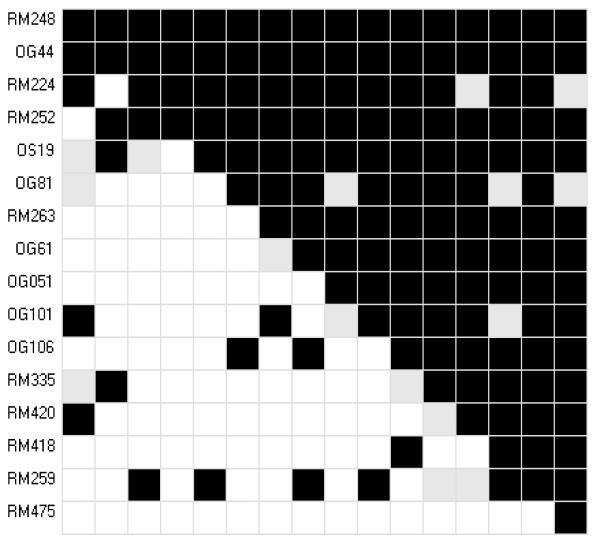
**Sampling effect in measurements of linkage disequilibrium between 16 microsatellite loci**. Sampling effect in measurements of linkage disequilibrium between 16 microsatellite loci. The upper triangle represents values of significance for the 485 accessions of the thematic collection; the lower triangle represents values of significance for the thematic core collection of 87 accessions (CCCorex). Black boxes indicate high significance (p < 0.0001); gray boxes indicate intermediate values (0.01 > p > 0.0001); white boxes indicate low levels of significance (p > 0.01).

Considering the entire collection of 485 accessions, the great majority of the pairwise comparisons between microsatellite loci had significant values for linkage disequilibrium (*r *^2 ^and *D*') (Figure 2). There was a change in the scenario when the analysis was performed with the 87 accessions that compose CC _corex_, with only 24% of the possible comparisons presenting significant values of linkage disequilibrium.

### Comparison of the methodologies used to define core collections

For comparative purposes, starting with the same set of DNA polymorphism at microsatellite loci, three other core collections were obtained by random sampling and through the use of the M strategy, utilizing the MStrat and Powercore programs (Table 1). When a collection of 87 samples generated by random sampling (replicated 100 times) was analyzed for comparative purposes (CC _random sampling_), the average value for gene diversity dropped to 0.647. This value is comparable to that of the thematic collection and significantly lower (*p *≤ 3.052 × 10 ^-5 ^in Wilcoxon's signed-rank test; *p *≤ 5.240 × 10 ^-5 ^in Student's *t *test) than that obtained for the CC _corex_. Also, the percentage of alleles from the thematic collection retained by the random sampling strategy dropped to 72.90% (Table 1).

The collection obtained when using the MStrat program (CC _MStrat_), is composed of 51 accessions representing 10.51% of the size of the thematic collection. CC _MStrat _contains 85.6% (196) of the alleles present in the thematic collection, and an average value of gene diversity of 0.739. The collection which was obtained by the Powercore program (CC _Powercore_) is composed of 82 accessions (16.90% of the size of the thematic collection), and contains 100% of the alleles present in the thematic collection and an average gene diversity value of 0.745.

As pointed out above, 100% of the alleles detected in the thematic collection were maintained by CC _Powercore_. CC _corex _and CC _MStrat _showed a different behavior in relation to the loss of alleles that were present in the thematic collection: of the initial set of 229 alleles in the thematic collection (Table 1), 21 were lost in the collection defined by the MStrat program (CC _MStrat_), but maintained by CC _corex_. However, other 24 alleles were lost by CC _corex_, but maintained by CC _MStrat_. Twelve alleles present in the thematic collection were lost in these the two collections. By setting up the boundaries of the thematic collection into two groups, as described by [18], it was observed that five out of the 21 alleles lost by CC _MStrat _were exclusive to one of the two groups (rare and specifically located alleles). When examining CC _corex_, it was observed that 15 out of the 24 alleles lost were exclusive. Five out of the 12 alleles lost in both collections were exclusive to one of the two groups.

### Use of quantitative data for the comparison of different methodologies for building core collections

The ANOVA of the quantitative data of the rice accessions that compose CC _corex _which were evaluated for drought tolerance in the field demonstrated that the best phenotypic data to be used to compare the core collection strategies under analysis would be the Weight of 100 Grains. A significant difference in the values of this variable between the accessions of the core collection (*p *< 0.01, *F *test, CV = 4.34%) was observed in the experiment. All other traits had no significant differences in their values between accessions in the core collection. For this trait, there was no loss of data and no significant differences between the replicated plots. Three core sub-collections of CC _corex _were obtained using the Corex (CSC _corex_), MStrat (CSC _MStrat_) and Powercore (CSC _Powercore_) methods, and compared to CSC _corex _and a core sub-collection based on the same random sampling procedure described previously (CSC _random sampling_).

A small change in the values of the standard deviation for the trait Weight of 100 grains in the three core sub-collections that were defined was observed (Table 2). The standard deviation in the core collection of 87 accessions (CC _corex_) had a value of 0.57. In CSC _corex _the value increased to 0.62, in CSC _Mstrat _to 0.64, in CSC _Powercore _to 0.61 and in CSC _random sampling _to 0.60. However, the Bartlett's test showed homogeneity between the values of the standard deviation in the core collection (CC _corex_) and the sub-collections (CSC _corex_, CSC _Mstrat_, CSC _Powercore _and CSC _random sampling) _(Table 2). Estimated mean values of *GD *for CSC _corex_, CSC _Mstrat_, CSC _Powercore _and CSC _random sampling _were not significantly different between sampling methodologies using the TEST_h_DIFF [33], the paired t test [25] and Wilcoxon's signed-rank test.

**Table 2:** Gene Diversity (*GD*), mean and standard deviation of the trait Weight of 100 Grains (g) of core sub-collections defined from a core collection (CC_corex_) using different sampling methodologies

Methodology	GD	Mean	Standard Deviation
CC_corex_	0.764^ns^	2.89	0.57 ^ns^
CSC_corex_	0.767 ^ns^	2.85	0.62 ^ns^
CSC_MStrat_	0.739 ^ns^	2.90	0.64 ^ns^
CSC_Powercore_	0.783 ^ns^	2.93	0.61 ^ns^
CSC_random sampling_	0.759 ^ns^	2.87	0.60 ^ns^

^ns^differences of GD values not significant between methodologies using the TEST_h_DIFF (Wealer, 2003), the paired t test (Nei, 1987) and Wilcoxon's signed-rank test; differences of Standard Deviation values not significant (Bartlett's test)

## Discussion

The use of sampling strategies for defining core collections based on genetic distance is not new. On the other hand, rare are the examples of automated procedures which would make it possible for this type of analysis to be carried out on a large scale, especially with the advent of high throughput genotyping methodologies now available. An approach for obtaining core collections using genetic distance data obtained from molecular marker analysis (Corex), allowing comparisons with randomly sampled collections and estimates of allele retention from the thematic collection has been proposed. The objective of the present study was to develop a thematic core collection that maintained at least 80% of the total number of alleles detected in a thematic collection showing genetic variability for drought tolerance. A core collection (CC _corex_) with 18% of the size of the reference collection was obtained (87 accessions out of a total of 485), containing 84.3% of the total number of alleles, and with significantly higher values of *GD *when compared to the thematic collection and with a collection of the same size based on random sampling (CC _random sampling_).

The comparison of allelic richness *versus *the size of the core collection shows a rapid increase in the number of alleles as the size of the core collection reaches approximately 80% of the total number of alleles detected in the thematic collection (Figure 1). The alleles represented in smaller collections are generally the most common ones; however, they are different between the accessions themselves, justifying this behavior. The curve then seems to reach a *plateau *since the presence of new low-frequency alleles would require samples of greater size for their proper detection (Figure 1). Effects of inbreeding were also detected in CC _corex_, but the average estimate of *F*_ST _decreased in relation to the thematic collection, demonstrating a moderate differentiation, although significant, between the groups that were inferred. The distribution of genetic diversity between the groups presented a similar behavior in relation to that observed in the thematic collection.

When compared to a collection of the same size, but which was created by random sampling (CC _random sampling_), CC _corex _had higher allele richness and a greater average *GD *value. This effect concerning the increase in the values of diversity in the core collection was reported by [11], but it is rarely verified or reported in studies concerning the development of core collections, where the main concern seems to be the representation of the genetic diversity present in large samples, and not its maximization. Two other core collections which were defined for comparative purposes using the M strategy (CC _Powercore _and CC _MStrat_) maintained a greater proportion of alleles (in the case of the Powercore program, with 100% of the alleles in 82 accessions) or equivalent, with a smaller number of accessions (in the case of the MStrat program, with 85.6% of alleles in 51 accessions). A comparison between the data banks of allele frequencies of the collections established using the different methodologies demonstrates that when there is a loss of alleles - as in CC _corex _and CC _Mstrat _- there doesn't appear to be a specific pattern for the type of allele that is lost in relation to their frequencies. It was noticed, for example, that of the 57 alleles that were lost in either of the two collections (CC _corex _and CC _MStrat_), only one had an intermediate frequency (0.05 < frequency < 0.30), which was lost in the CC _MStrat _collection. All of the other alleles are of a very low frequency, with values that vary between 0.0011 (allele 89 of the locus OG81 and allele 70 of the locus RM248) and 0.0412 (allele 83 of the locus OG81). Nevertheless, there was some variation in relation to the type of allele that was lost concerning its location in different populations defined *a priori *in the thematic collection. In this case, CC _corex _lost more localized and low frequency alleles, maintaining a greater proportion of alleles present in the two groups of the thematic collection. On the other hand, CC _MStrat _lost a greater proportion of this last type of allele, prioritizing rare and localized alleles.

**Figure 3:**
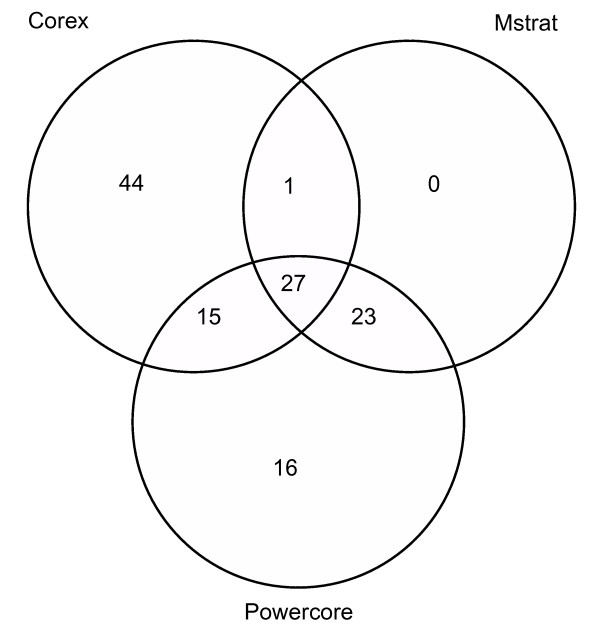
**Analysis of the coincidence of accessions sampled by the three methods (Corex, MStrat and Powercore) used for building core collections**.

The analysis of the coincidence of sampled accessions by the three methods (Corex, Powercore, and MStrat) indicates that (Figure 3): (a) as a whole, when examining 128 distinct accessions which were selected by the three methods, only 21% of the total sample are common to all the methods; 22% are common to CC _corex _and CC _MStrat_; 33% are common to CC _corex _and CC _Powercore_; and 39% are common to CC _Powercore _and CC _MStrat_; (b) considering only the accessions which were sampled using the Powercore and MStrat methods, with the exception of one accession, all of the other 50 accessions which were sampled were common to the two methods. This indicates that the algorithms used for these two methods are very similar; (c) the Corex method presents 34% of the unique accessions, which were sampled only by this method, while the Powercore method sampled 16 unique accessions and MStrat had no unique accessions, i.e., having a smaller size, all of its accessions were also selected by the other methods (Figure 3).

It seems that this type of behavior is expected based on the apparent rationale of each methodology. The M Strategy examines all possible core collections and selects those that maximize the number of alleles observed at each locus. Previous studies describe the efficiency of this sampling technique in cases where the species in question is autogamous (which is the case for rice), or in the absence of migration [7]. The strategy would favor the capture of spread out alleles (those that are not specifically localized in a population) which occur with a low frequency, and also population specific alleles, which occur with a higher frequency.

The core collection defined by the method described in this study (Corex - maximization of genetic distance) presented an average value of gene diversity that was greater than the two collections obtained by the M strategy (MStrat and Powercore). This difference, however, is not statistically significant. Two slightly distinct behaviors were observed in relation to the number of alleles and gene diversity: the methodology based upon genetic distance maximizes values of gene diversity in a sample size which is suitable to that traditionally defined for core collections, while the two collections defined by the M strategy more efficiently maximize the presence of alleles of the thematic collection within the core collection, whether it is a collection of equal (82 accessions in the Powercore program) or smaller size (51 accessions in the MStrat program). However, the difference represented in values of gene diversity is not significant.

The increase in the average gene diversity value in CC _corex _reflects the true nature of the selection criterion utilized during the process. The coefficient of distance which was used - *shared allele distance *- takes into consideration the proportion of alleles shared between two samples: if two individuals share 6 alleles out of a total of 10 allele copies, the coefficient of distance between them is 1 - (6/10) = 0.4. Thus, the Corex algorithm, upon using the values for *shared allele distance *as a criterion for classification of accessions, inserts those accessions with a lower probability of having alleles in common into the core collection. The coefficient for gene diversity, frequently called expected heterozygosity in allogamous species, or average heterozygosity, represents the probability that two alleles which are randomly taken from the population are different, and it is calculated based on the reciprocity of the sum of the square of the allelic frequency for each loci. For an autogamous species, the gene diversity is a more appropriate measure of variation than heterozygosity, due to the occurrence of few heterozygotes and the presence of several different types of homozygotes. Thus, accessions included in the core collection which share low proportions of alleles would reflect a greater probability that two alleles taken randomly from the population would be different - in other words, greater values in gene diversity. Hence, sampling based on genetic distance aims to produce a core collection which is useful for breeding. Its focus is not on obtaining rare and highly restricted alleles which maximize the total diversity of the core collection, but rather to maximize the level of representation of the genetic diversity in the collection.

An approach based on genetic distances allows for the use of data generated from any type of genetic information, whether it came from dominant molecular markers, morpho-agronomic traits, or even a coefficient of distance which combines the two types of information. As is the case with the M strategy, the approach based on genetic distance also makes it possible to take samples without the need of an *a priori *stratification of the thematic collection (an initial stage in various methodologies which have been proposed for sampling of core collections) - however, this does not eliminate the possibility of using this methodology together with the stratification of accessions in groups that are genetically similar (if the stratification is a theme, or a trait, as the first step to list the accessions which will be considered for analysis). The use of the technique proposed here in other databases which can also be sampled by the M strategy may indicate if the effect of greater maximization of gene diversity is inherent to the sampling technique or if it is dependent upon the thematic collection being analyzed.

Investigations about the extension of linkage disequilibrium in plants have been a topic of great interest recently, mainly due to its impact in planning studies dealing with the association genetics for gene discovery, as well as the use of genome wide selection in breeding programs [36]. Both biological factors and evolutionary history affect the extension of linkage disequilibrium, which can be caused by a physical linkage between loci, or by demographic history (bottlenecks, migration, and admixture**), **genomic history (reproduction, recombination, mutation) and selection. In the present study, when the complete set of 485 accessions was analyzed (thematic population), there was evidence of disequilibrium between unlinked microsatellite loci in nearly all the pairwise comparisons tested (Figure 2). However, a significant reduction in spurious detection of linkage disequilibrium was verified in the core collection defined by genetic distance (CC _corex_). Spurious detection in the thematic population was possibly caused by the high frequency of similar genotypes in individuals that are genetically more related. When diversity is maximized during the process of establishing a core collection, with the elimination of accessions that are genetically more related, independence is observed in the majority of the pairwise comparisons between loci. Figure 2 graphically depicts these sampling effects on linkage disequilibrium for CC _corex_. The boxes painted in black indicate values of linkage disequilibrium that are highly significant, present mainly in the superior portion of the graph, which represents the complete set of 485 accessions (thematic collection). In the inferior portion of the graph, the majority of the pairwise comparisons between marker loci indicate independence in CC _corex_, represented by the white boxes. Similar behavior was observed in wheat and grapevine [37,38]. It is worthwhile noticing that the sample size also has a direct effect on this behavior and may increase spurious detection of linkage disequilibrium. Thus, the sampling methodology should also take this effect into consideration.

An attempt to compare the methods for building sub core collections from the core collection of 87 accessions (CC _corex_) using phenotypic data evaluated in the field as a measure of their diversity demonstrated that for an independent agronomic trait (Weight of 100 Grains), where significant variability exists between the accessions, an increase in the variability (measured by values of standard deviation) occurs in the core sub-collections obtained using the Corex, MStrat and Powercore methodologies (Table 2). Nevertheless, this increase is not significant (Table 2). When comparing the genetic diversity of the core sub-collections CSC _corex_, CSC _MStrat_, CSC _Powercore _and CSC _random sampling_, it was observed that there was no significant increase in *GD *in the resulting core sub-collections (Table 2). All significance tests, when they were compared to the core collection obtained by the Corex program (CC _corex_), and the core sub-collection generated randomly from it, generated *GD *values that were significantly similar (Table 2).

## Conclusion

As a consequence of the occurrence of increasingly large germplasm collections of certain species, especially grasses, core collections of a large number of accessions have been developed which, usually, are of limited use by breeding programs. However, large germplasm collections allow for selection of groups of accessions with maximized genetic variability for specific traits of interest. Those groups compose thematic collections, which can be used to develop thematic core collections, a relatively small set of genetically divergent accessions showing variability for a specific trait, which would be of strategic use by breeding programs.

Building of a thematic core collection was here defined by prior selection of accessions which are diverse for the trait of interest (thematic collection), and then by pairwise genetic distances, estimated by DNA polymorphism analysis at molecular marker loci. This showed to be a methodology able to generate core collections which potentially reflect the maximum allele richness with the smallest sample size from a thematic collection. That is to say, for different, complex traits, different thematic core collections would be defined from large collections.

Thus, a germplasm collection would not only have a defined core collection for the entire collection, as has been proposed [29] and tested [6,7,15,16], but also various thematic core collections of smaller sizes focusing on different traits that are of interest to plant breeding programs. In this study, we used as an example the development of a thematic core collection for drought tolerance in rice. It is expected that such thematic collections increase the use of germplasm by breeding programs and facilitate the study of the traits under consideration. The definition of a core collection to study drought resistance is a valuable contribution towards the understanding of the genetic control and the physiological mechanisms involved in water use efficiency in plants.

## Authors' contributions

MPF performed all computational as well as statistical analyses and drafting of the manuscript. PHNR selected and provided the plant material used in this study. MEF conceived and supervised the study, and edited the manuscript. All authors read and approved the final manuscript.
